# *N*-(2-Chloro­eth­yl)pyrazine-2-carboxamide

**DOI:** 10.1107/S1600536810041656

**Published:** 2010-10-23

**Authors:** Camilo H. da Silva Lima, Marcus V. N. de Souza, Solange M. S. V. Wardell, James L. Wardell, Edward R. T. Tiekink

**Affiliations:** a Fundaçao Oswaldo Cruz, Instituto de Tecnologia em Fármacos - Farmanguinhos, R. Sizenando Nabuco 100, Manguinhos, 21041-250, Rio de Janeiro, RJ, Brazil; b CHEMSOL, 1 Harcourt Road, Aberdeen AB15 5NY, Scotland; c Centro de Desenvolvimento Tecnológico em Saúde (CDTS), Fundação Oswaldo Cruz (FIOCRUZ), Casa Amarela, Campus de Manguinhos, Av. Brasil 4365, 21040-900, Rio de Janeiro, RJ, Brazil; d Department of Chemistry, University of Malaya, 50603 Kuala Lumpur, Malaysia

## Abstract

In the title mol­ecule, C_7_H_8_ClN_3_O, the pyrazine and amide groups are almost co-planar [N—C—C—N torsion angle = −2.4 (2) °], a conformation stabilized by an intra­molecular N—H⋯N hydrogen bond. The chloro­ethyl group lies out of the plane [N—C—C—Cl = −65.06 (17) °]. In the crystal, the presence of N—H⋯N hydrogen bonds leads to the formation of a *C*(6) supra­molecular chain along the *b* axis. The carbonyl-O atom accepts two C—H⋯O inter­actions. These, plus Cl⋯Cl short contacts [3.3653 (6) Å], consolidate the packing of the chains in the crystal.

## Related literature

For the anti­mycobacterial activity of pyrazinamide, see: Chaisson *et al.* (2002 ▶); Gordin *et al.* (2000 ▶); de Souza (2006 ▶). For structural studies on pyrazinamide derivatives; see: Wardell *et al.* (2008 ▶); Baddeley *et al.* (2009 ▶); Howie *et al.* (2010*a* ▶,*b* ▶,*c* ▶,*d* ▶).
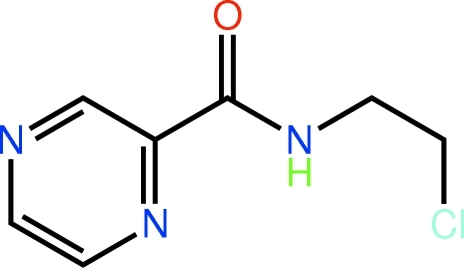


## Experimental

### 

#### Crystal data

C_7_H_8_ClN_3_O
*M* *_r_* = 185.61
Monoclinic, 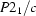

*a* = 4.4639 (2) Å
*b* = 10.6865 (6) Å
*c* = 17.3583 (9) Å
β = 93.028 (3)°
*V* = 826.89 (7) Å^3^
*Z* = 4
Mo *K*α radiation
μ = 0.41 mm^−1^
*T* = 120 K
0.28 × 0.18 × 0.03 mm

#### Data collection

Nonius KappaCCD diffractometer
Absorption correction: multi-scan (*SADABS*; Sheldrick, 2007 ▶) *T*_min_ = 0.631, *T*_max_ = 0.746
16245 measured reflections
1867 independent reflections
1628 reflections with *I* > 2σ(*I*)
*R*_int_ = 0.044

#### Refinement

*R*[*F* ^2^ > 2σ(*F* ^2^)] = 0.036
*wR*(*F* ^2^) = 0.110
*S* = 1.15
1867 reflections
112 parameters
1 restraint
H atoms treated by a mixture of independent and constrained refinement
Δρ_max_ = 0.23 e Å^−3^
Δρ_min_ = −0.37 e Å^−3^

### 

Data collection: *COLLECT* (Hooft, 1998 ▶); cell refinement: *DENZO* (Otwinowski & Minor, 1997 ▶) and *COLLECT*; data reduction: *DENZO* and *COLLECT*; program(s) used to solve structure: *SHELXS97* (Sheldrick, 2008 ▶); program(s) used to refine structure: *SHELXL97* (Sheldrick, 2008 ▶); molecular graphics: *ORTEP-3* (Farrugia, 1997 ▶) and *DIAMOND* (Brandenburg, 2006 ▶); software used to prepare material for publication: *publCIF* (Westrip, 2010 ▶).

## Supplementary Material

Crystal structure: contains datablocks general, I. DOI: 10.1107/S1600536810041656/hb5682sup1.cif

Structure factors: contains datablocks I. DOI: 10.1107/S1600536810041656/hb5682Isup2.hkl

Additional supplementary materials:  crystallographic information; 3D view; checkCIF report

## Figures and Tables

**Table 1:** Hydrogen-bond geometry (Å, °)

*D*—H⋯*A*	*D*—H	H⋯*A*	*D*⋯*A*	*D*—H⋯*A*
N1—H1*n*⋯N2	0.87 (2)	2.34 (2)	2.7162 (19)	107 (1)
N1—H1*n*⋯N3^i^	0.87 (2)	2.33 (2)	3.146 (2)	156 (2)
C5—H5⋯N2^ii^	0.95	2.60	3.212 (2)	123
C7—H7*A*⋯O1^iii^	0.99	2.44	3.180 (2)	131
C7—H7*B*⋯O1^iv^	0.99	2.42	3.337 (2)	153

Symmetry codes: (i) 
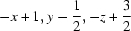
; (ii) 
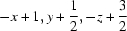
; (iii) 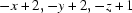; (iv) 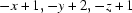.

**Fig. 1.**
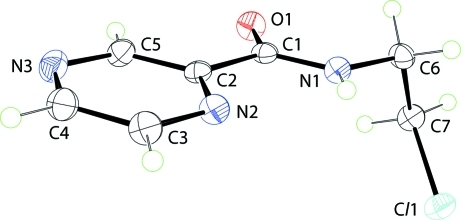
The molecular structure of (I) showing displacement ellipsoids at the 50% probability level.

**Fig. 2.**
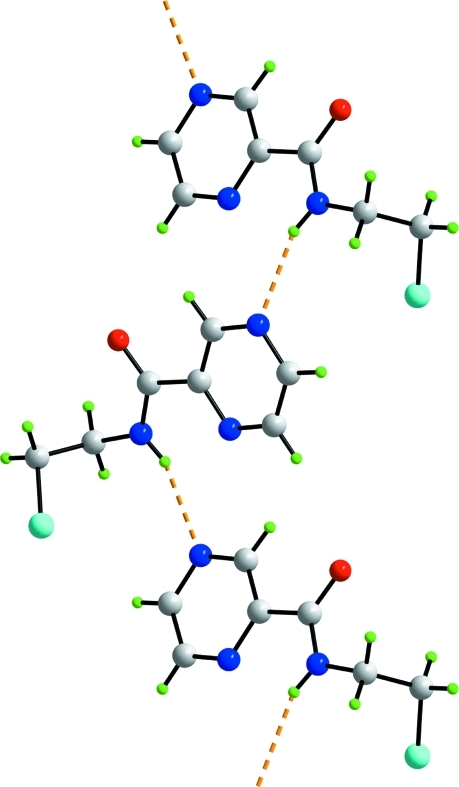
Supramolecular chain in (I) aligned along the b axis. The N—H···N hydrogen bonds are shown as blue dashed lines.

**Fig. 3.**
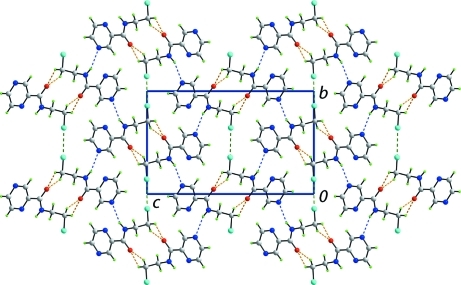
A view in projection down the a axis of the crystal packing in (I). The N—H···N hydrogen bonds, and C—H···O and Cl···Cl contacts are shown as blue, orange and green dashed lines, respectively.

## supplementary crystallographic information

### Comment

Pyrazinamide has well known anti-mycobacterial activity and is the one of the most important drugs used in tuberculosis treatment (Chaisson *et al.*, 2002; Gordin *et al.*, 2000; de Souza, 2006). In continuation of our studies on pyrazinamide derivatives (Wardell *et al.*, 2008; Baddeley *et al.*, 2009; Howie *et al.*, 2010*a*, 2010*b*, 2010*c*, 2010*d*), we report the structure of title compound, (I).

The pyrazine and amide groups are co-planar as seen in the value of the N1—C1—C2—N2 torsion angle of -2.4 (2) °, a conformation stabilized by an intramolecular N1—H···N2 hydrogen bond, Table 1. The ethyl group lies out of the plane through the rest of the molecule as seen in the N1—C6—C7—Cl1 torsion angle of -65.06 (17) °. The carbonyl-O1 lies to the opposite side of the molecule occupied by the amide and chlorido atoms.

In the crystal packing, the most prominent interactions are hydrogen bonding interactions of the type N—H···N, Table 1, which lead to a supramolecular chain along the screw axis, Fig. 2. The chains are connected into the 3-D structure by C—H···O interactions, involving the bifurcated carbonyl-O atom interacting with two methylene-H atoms, Table 1, and Cl···Cl contacts [Cl1···Cl1^i^ = 3.3653 (6) Å for *i*: 1 - *x*, 1 - *y*, 1 - *z*], Fig. 3.

### Experimental

The title compound was prepared by refluxing a mixture of thionyl chloride (1 ml) and *N*-(2-chloroethyl)pyrazine-2-carboxamide (0.2 g), obtained from methyl 2-pyrazinecarboxylate, ethanolamine and triethylamine. After 6 h, the excess thionyl chloride was removed under reduced pressure, the residue extracted into ethyl acetate (20 ml) and washed with saturated sodium bicarbonate solution (60 ml). The organic phase was dried over Na_2_SO_4_ and concentrated under reduced pressure to afford title compound, yield: 70%; m. pt.: 384–386 K. The crystals used in the structure determination were grown from EtOH solution.

^1^H NMR (200 MHz, DMSO-d6) δ: 9.19 (1*H*, s, H3), 9.12 (1*H*, s, NH), 8.88 (1*H*, s, H6), 8.74 (1*H*, s, H5), 3.76–3.63 (4*H*, m, CH_2_CH_2_Cl). ^13^C NMR (50 MHz, DMSO-d6) δ: 153.8, 138.4, 135.2, 134.3, 134.1, 33.6, 31.6 p.p.m.. MS/ESI: [M—H]: 184.

### Refinement

The C-bound H atoms were geometrically placed (C–H = 0.95–0.99 Å) and refined as riding with *U*_iso_(H) = 1.2*U*_eq_(C). The N-bound H atom was located from a difference map and refined with the distance restraint N–H = 0.88±0.01 Å, and with *U*_iso_(H) = 1.2*U*_eq_(N).

### Crystal data

**Table:**

C_7_H_8_ClN_3_O	*F*(000) = 384
*M**_r_* = 185.61	*D*_x_ = 1.491 Mg m^−^^3^
Monoclinic, *P*2_1_/*c*	Mo *K*α radiation, λ = 0.71073 Å
Hall symbol: -P 2ybc	Cell parameters from 25954 reflections
*a* = 4.4639 (2) Å	θ = 2.9–27.5°
*b* = 10.6865 (6) Å	µ = 0.41 mm^−^^1^
*c* = 17.3583 (9) Å	*T* = 120 K
β = 93.028 (3)°	Prism, colourless
*V* = 826.89 (7) Å^3^	0.28 × 0.18 × 0.03 mm
*Z* = 4	

### Data collection

**Table:**

Nonius KappaCCD diffractometer	1867 independent reflections
Radiation source: Enraf Nonius FR591 rotating anode	1628 reflections with *I* > 2σ(*I*)
10 cm confocal mirrors	*R*_int_ = 0.044
Detector resolution: 9.091 pixels mm^-1^	θ_max_ = 27.5°, θ_min_ = 3.0°
φ and ω scans	*h* = −5→5
Absorption correction: multi-scan (*SADABS*; Sheldrick, 2007)	*k* = −13→13
*T*_min_ = 0.631, *T*_max_ = 0.746	*l* = −22→22
16245 measured reflections	

### Refinement

**Table:**

Refinement on *F*^2^	Primary atom site location: structure-invariant direct methods
Least-squares matrix: full	Secondary atom site location: difference Fourier map
*R*[*F*^2^ > 2σ(*F*^2^)] = 0.036	Hydrogen site location: inferred from neighbouring sites
*wR*(*F*^2^) = 0.110	H atoms treated by a mixture of independent and constrained refinement
*S* = 1.15	*w* = 1/[σ^2^(*F*_o_^2^) + (0.0577*P*)^2^ + 0.3276*P*] where *P* = (*F*_o_^2^ + 2*F*_c_^2^)/3
1867 reflections	(Δ/σ)_max_ < 0.001
112 parameters	Δρ_max_ = 0.23 e Å^−^^3^
1 restraint	Δρ_min_ = −0.37 e Å^−^^3^

### Special details

**Table:**

Geometry. All s.u.'s (except the s.u. in the dihedral angle between two l.s. planes) are estimated using the full covariance matrix. The cell s.u.'s are taken into account individually in the estimation of s.u.'s in distances, angles and torsion angles; correlations between s.u.'s in cell parameters are only used when they are defined by crystal symmetry. An approximate (isotropic) treatment of cell s.u.'s is used for estimating s.u.'s involving l.s. planes.
Refinement. Refinement of *F*^2^ against ALL reflections. The weighted *R*-factor *wR* and goodness of fit *S* are based on *F*^2^, conventional *R*-factors *R* are based on *F*, with *F* set to zero for negative *F*^2^. The threshold expression of *F*^2^ > 2σ(*F*^2^) is used only for calculating *R*-factors(gt) *etc*. and is not relevant to the choice of reflections for refinement. *R*-factors based on *F*^2^ are statistically about twice as large as those based on *F*, and *R*- factors based on ALL data will be even larger.

### Fractional atomic coordinates and isotropic or equivalent isotropic displacement parameters (Å^2^)

**Table:**

	*x*	*y*	*z*	*U*_iso_*/*U*_eq_	
Cl1	0.65306 (10)	0.64380 (4)	0.49830 (2)	0.02780 (17)	
O1	0.7056 (3)	1.04630 (12)	0.60138 (7)	0.0247 (3)	
N1	0.8112 (3)	0.84349 (13)	0.63161 (8)	0.0195 (3)	
H1n	0.785 (5)	0.7824 (15)	0.6637 (10)	0.028*	
N2	0.4415 (3)	0.85341 (14)	0.75082 (8)	0.0222 (3)	
N3	0.1647 (4)	1.07915 (15)	0.78848 (9)	0.0275 (4)	
C1	0.6754 (4)	0.95343 (16)	0.64249 (9)	0.0182 (3)	
C2	0.4763 (4)	0.95775 (15)	0.70976 (9)	0.0180 (3)	
C3	0.2635 (4)	0.86226 (18)	0.80989 (10)	0.0249 (4)	
H3	0.2273	0.7897	0.8396	0.030*	
C4	0.1292 (4)	0.97467 (19)	0.82923 (10)	0.0264 (4)	
H4	0.0087	0.9772	0.8727	0.032*	
C5	0.3352 (4)	1.06922 (17)	0.72769 (10)	0.0229 (4)	
H5	0.3607	1.1403	0.6959	0.028*	
C6	1.0072 (4)	0.82477 (17)	0.56832 (9)	0.0212 (4)	
H6A	1.1499	0.7565	0.5821	0.025*	
H6B	1.1252	0.9020	0.5613	0.025*	
C7	0.8395 (4)	0.79260 (17)	0.49280 (10)	0.0232 (4)	
H7A	0.9823	0.7900	0.4511	0.028*	
H7B	0.6894	0.8586	0.4799	0.028*	

### Atomic displacement parameters (Å^2^)

**Table:**

	*U*^11^	*U*^22^	*U*^33^	*U*^12^	*U*^13^	*U*^23^
Cl1	0.0327 (3)	0.0252 (3)	0.0255 (3)	−0.00579 (17)	0.00184 (18)	−0.00416 (17)
O1	0.0294 (7)	0.0222 (7)	0.0229 (6)	−0.0018 (5)	0.0045 (5)	0.0058 (5)
N1	0.0221 (7)	0.0200 (8)	0.0164 (7)	−0.0010 (6)	0.0025 (5)	0.0017 (5)
N2	0.0248 (8)	0.0230 (8)	0.0190 (7)	−0.0010 (6)	0.0023 (6)	0.0019 (6)
N3	0.0312 (8)	0.0271 (9)	0.0245 (8)	−0.0003 (6)	0.0048 (6)	−0.0055 (6)
C1	0.0173 (7)	0.0210 (8)	0.0163 (7)	−0.0040 (6)	−0.0011 (6)	−0.0017 (6)
C2	0.0190 (8)	0.0194 (8)	0.0152 (7)	−0.0036 (6)	−0.0014 (6)	−0.0013 (6)
C3	0.0293 (9)	0.0279 (10)	0.0179 (8)	−0.0026 (7)	0.0042 (7)	0.0026 (7)
C4	0.0282 (9)	0.0333 (10)	0.0181 (8)	−0.0029 (8)	0.0047 (7)	−0.0031 (7)
C5	0.0272 (9)	0.0211 (9)	0.0206 (8)	−0.0024 (7)	0.0028 (7)	−0.0015 (7)
C6	0.0189 (8)	0.0253 (9)	0.0199 (8)	−0.0018 (7)	0.0046 (6)	−0.0006 (7)
C7	0.0273 (9)	0.0237 (9)	0.0190 (8)	−0.0025 (7)	0.0044 (7)	0.0011 (7)

### Geometric parameters (Å, °)

**Table:**

Cl1—C7	1.7997 (19)	C2—C5	1.390 (2)
O1—C1	1.234 (2)	C3—C4	1.391 (3)
N1—C1	1.340 (2)	C3—H3	0.9500
N1—C6	1.454 (2)	C4—H4	0.9500
N1—H1n	0.870 (10)	C5—H5	0.9500
N2—C3	1.334 (2)	C6—C7	1.514 (2)
N2—C2	1.337 (2)	C6—H6A	0.9900
N3—C4	1.336 (3)	C6—H6B	0.9900
N3—C5	1.338 (2)	C7—H7A	0.9900
C1—C2	1.505 (2)	C7—H7B	0.9900
			
C1—N1—C6	121.46 (14)	C3—C4—H4	119.0
C1—N1—H1N	119.4 (14)	N3—C5—C2	121.86 (17)
C6—N1—H1N	119.1 (14)	N3—C5—H5	119.1
C3—N2—C2	116.20 (15)	C2—C5—H5	119.1
C4—N3—C5	116.09 (16)	N1—C6—C7	113.30 (14)
O1—C1—N1	124.03 (15)	N1—C6—H6A	108.9
O1—C1—C2	120.73 (15)	C7—C6—H6A	108.9
N1—C1—C2	115.24 (14)	N1—C6—H6B	108.9
N2—C2—C5	121.91 (15)	C7—C6—H6B	108.9
N2—C2—C1	118.55 (15)	H6A—C6—H6B	107.7
C5—C2—C1	119.53 (15)	C6—C7—Cl1	111.32 (12)
N2—C3—C4	121.91 (17)	C6—C7—H7A	109.4
N2—C3—H3	119.0	Cl1—C7—H7A	109.4
C4—C3—H3	119.0	C6—C7—H7B	109.4
N3—C4—C3	121.95 (16)	Cl1—C7—H7B	109.4
N3—C4—H4	119.0	H7A—C7—H7B	108.0
			
C6—N1—C1—O1	−0.6 (2)	C2—N2—C3—C4	−2.0 (3)
C6—N1—C1—C2	179.20 (13)	C5—N3—C4—C3	0.4 (3)
C3—N2—C2—C5	0.1 (2)	N2—C3—C4—N3	1.8 (3)
C3—N2—C2—C1	179.85 (15)	C4—N3—C5—C2	−2.3 (3)
O1—C1—C2—N2	177.44 (15)	N2—C2—C5—N3	2.2 (3)
N1—C1—C2—N2	−2.4 (2)	C1—C2—C5—N3	−177.58 (15)
O1—C1—C2—C5	−2.8 (2)	C1—N1—C6—C7	−83.3 (2)
N1—C1—C2—C5	177.37 (15)	N1—C6—C7—Cl1	−65.06 (17)

### Hydrogen-bond geometry (Å, °)

**Table:**

*D*—H···*A*	*D*—H	H···*A*	*D*···*A*	*D*—H···*A*
N1—H1n···N2	0.87 (2)	2.34 (2)	2.7162 (19)	107 (1)
N1—H1n···N3^i^	0.87 (2)	2.33 (2)	3.146 (2)	156 (2)
C5—H5···N2^ii^	0.95	2.60	3.212 (2)	123
C7—H7A···O1^iii^	0.99	2.44	3.180 (2)	131
C7—H7B···O1^iv^	0.99	2.42	3.337 (2)	153

Symmetry codes: (i) −*x*+1, *y*−1/2, −*z*+3/2; (ii) −*x*+1, *y*+1/2, −*z*+3/2; (iii) −*x*+2, −*y*+2, −*z*+1; (iv) −*x*+1, −*y*+2, −*z*+1.
